# Incorporation of *Glycine max Merrill* Extract into Layered Double Hydroxide through Ion-Exchange and Reconstruction

**DOI:** 10.3390/nano9091262

**Published:** 2019-09-05

**Authors:** Do-Gak Jeung, Hyoung-Jun Kim, Jae-Min Oh

**Affiliations:** Department of Energy and Materials Engineering, Dongguk University-Seoul, Seoul 04620, Korea

**Keywords:** layered double hydroxide, *Glycine max Merrill* extract, incorporation method, release, alkaline phosphatase activity

## Abstract

We incorporated extract of *Glycine max Merrill* (GM), which is generally known as soybean, into a layered double hydroxide (LDH) nanostructure through two different methods, ion-exchange and reconstruction. Through X-ray diffraction, field-emission scanning electron microscopy, and zeta-potential measurement, GM moiety seemed to be simply attached on the surface of LDH by ion-exchange process, while the extract could be incorporated in the inter-particle pore of LDHs by reconstruction reaction. The quantification exhibited that both incorporation method showed comparable extract loading capacity of 15.6 wt/wt% for GM-LDH hybrid prepared by ion-exchange (GML-I) and 18.6 wt/wt% for GM-LDH hybrid by reconstruction (GML-R). On the other hand, bioactive substance in both GM-LDH hybrids, revealed that GML-R has higher daidzein content (0.0286 wt/wt%) compared with GML-I (0.0108 wt/wt%). According to time-dependent daidzein release, we confirmed that GML-R showed pH dependent daidzein release; a higher amount of daidzein was released in pH 4.5 physiological condition than in pH 7.4, suggesting the drug delivery potential of GML-R. Furthermore, alkaline phosphatase activity and collagen fiber formation on human osteoblast-like MG-63 cells displayed that GML-R had superior possibility of osteoblast differentiation than GML-I. From these results, we concluded that reconstruction method was more effective for extract incorporation than ion-exchange reaction, due to its pH dependent release property and alkaline phosphatase activity.

## 1. Introduction

Layered double hydroxide (LDH), which has chemical formula of M(II)_1−x_ M(III)_x_(OH)_2_(A^n−^)_x/n_·mH_2_O (M(II): divalent metal cation, M(III): trivalent metal cation, A^n−^: interlayer anion, 0.2 < x < 0.4), is composed of positively charged metal hydroxide sheets and charge compensating interlayer anions [1,2]. It has various properties such as biocompatibility, biological inertness, and anion exchange capacity [3,4,5], which allowed LDH to have great potential as a drug delivery carrier [4]. Furthermore, positively charged LDH is easily attached to a negatively charged cell membrane, and enters the cell in facilitated manner [6,7]. Additionally, it was reported that LDH with particle size from 50 to 200 nm had advantage in cellular uptake by specific clathrin-mediated endocytosis [8]. In addition to the effective cellular uptake property, LDH can enhance drug effect by protecting payloads and by releasing them in a controlled manner [9,10,11]. In this regard, many researchers have tried to incorporate various bioactive anionic species such as anticancer drugs, antioxidants, antibiotics, anti-inflammatory agents, vitamins, DNA, and even natural extract into LDH [9,12,13,14,15,16,17].

There are three possible chemical routes to introduce bioactive substances to size-controlled pristine LDHs: ion-exchange, reconstruction, and exfoliation-reassembly. In ion-exchange method, anionic molecules are adsorbed on the surface and in the gallery space of LDH through concentration gradient [18,19]. In the reconstruction process, layered double oxide (LDO), which is a calcined form of LDH, recovers its original LDH structure with intended anionic species when treated with water and the anionic moiety together [20]. In the exfoliation-reassembly reaction, LDH layers are first delaminated and then reassembled in the presence of intended anions [21]. However, the exfoliation-reassembly process obviously has limitation in biological application as the delaminated process often requires a toxic solvent like formamide [22,23].

Natural plant extract has long been studied in therapeutic application due to the biologically-active phytochemicals in the extract [24,25,26]. Jiang et al. reported that ethanol extract of *Angelica gigas* Nakai enhanced chemoprevention and chemotherapeutic properties against prostate cancer [27]. If natural plant extract was incorporated with a drug carrier such as LDH, the obtained hybrids would protect the activity of extract against the external harsh environment, facilitate drug delivery into the target cell, and release therapeutic components in a controlled manner [9,28]. At this stage, we noticed that the incorporation method would affect the biological behaviors of extract-LDH hybrids. As natural plant extract contains many substances ranging from single molecules to large bio-polymers [29], extract-LDH hybrids may exhibit different biological behavior depending on incorporation methods. It is easily expected that different incorporation routes provide both LDH host and guest species with a different chemical environment, resulting in different physicochemical properties of hybrids. In this study, therefore, we tried to prepare extract-LDH in two different methods. Then, the effect of hybridization between LDH and extract as well as the difference between incorporation methods would be investigated.

As a model for the extract part, soybean of which the scientific name is *Glycine max Merrill*, was selected. Soybean is one of the most consumed plants in the world and it contains various bioactive substances like phenolic acids, flavonoids, and isoflavonoids which show a therapeutic effect on cardiovascular disease, Alzheimer’s disease, and osteoporosis [30,31]. Especially, daidzein (DZ), one of the isoflavonoid derivatives in *Glycine max Merrill* can accelerate bone regeneration [32]. Thus, we can verify the difference of physicochemical property as well as biological activity of *Glycine max Merrill* extract (GM) incorporated LDH depending on incorporation methods.

In this study, we synthesized a hybrid of GM and LDH by two different methods, ion-exchange and reconstruction. The same pristine LDH was utilized for both reactions, in order to exclude any possible experimental errors except incorporation methods themselves. The differences in crystal structure, morphology, size, and surface charge of both hybrids were investigated using X-ray diffraction patterns, field-emission scanning electron microscopic images, and zeta-potential values. The chemical formula and GM content were investigated by thermogravimetry and elemental analysis. To determine the content of DZ, a bioactive moiety in GM, high performance liquid chromatography was carried out. Then, time-dependent release of DZ from hybrids were examined in physiological solution with neutral and slightly acidic pH. Furthermore, the biological activity of the hybrid was evaluated through cytotoxicity assay and alkaline phosphatase activity test.

## 2. Materials and Methods

### 2.1. Materials

Magnesium nitrate hexahydrate (Mg(NO_3_)_2_·6H_2_O) was purchased from Junsei Chemical Co., LTD. (Tokyo, Tokyo-do, Japan). Aluminum nitrate nonahydrate (Al(NO_3_)_3_·9H_2_O), the standard daidzein (DZ), triton-X100, and p-nitrophenyl phosphate liquid substance system (p-NPP) were obtained from Sigma-Aldrich Co. LLC. (St. Louis, MO, USA). Sodium hydroxide (NaOH), sodium triacetate, acetic acid, ethanol, and 1,1,1,3,3,3-hexamethyldisilazane were acquired form Daejung Chemicals & Metals Co. LTD. (Siheung, Gyeonggi-do, South Korea). Phosphate buffered saline (PBS) was purchased from Lonza, Ltd. (Basel, Basel-Suadt, Switzerland). Fetal bovine serum (FBS) was acquired from Thermo Fisher Scientific, Inc. (Waltham, MA, USA). Penicillin-streptomycin (100×), trypsin-EDTA, Dulbecco modified eagle medium (DMEM), Hank’s balanced salt solution (HBSS), and Trypan blue 0.4% were obtained from Welgene, Inc. (Gyeongsan, Gyeongsangbuk-do, South Korea). Cell proliferation reagent (WST-1) was obtained from F. Hoffmann-La Roche, Ltd. (Basel, Basel-Suadt, Switzerland). Methanol and acetonitrile (ACN) were purchased from Burdick & Jackson^®^ (Muskegon, MI, USA). Dimethyl sulfoxide (DMSO) and 4-paraformaldehyde were acquired from Biosesang Co. (Sungnam, Gyeonggi-do, South Korea). Protein assay dye reagent concentrate was obtained from Bio-Rad Lab, Inc. (Hercules, CA, USA). All reagents were utilized without further purification. 

### 2.2. Extraction of Glycine max Merrill

In order to obtain *Glycine max Merrill* extract (GM), first, 1 kg of *Glycine max Merrill* was mixed with 2 L of 100% methanol and then vigorously stirred for 24 h at room temperature. Then, methanol was evaporated using rotary vacuum evaporator at 60 °C. Thus, the obtained solid part was re-dissolved in DMSO for further reaction. The content of DZ in GM was determined by high performance liquid chromatography (HPLC, Yonglin YL9100 Series, Anyang, Gyeonggi-do, South Korea). The C-18 column, mixed solvent mobile phase (methanol:acetate buffer solution:ACN = 1:7:2), 1.0 mL/min flow rate, and UV-vis detector with a wavelength of 270 nm were applied for the chromatography. 

### 2.3. Preparation of GM-LDH Hybrids by Two Incorporation Routes

Pristine LDH with homogeneous particle size was prepared by conventional co-precipitation and hydrothermal method [33]. The mixed metal solution of Mg(NO_3_)_2_·6H_2_O (0.1575 M) and Al(NO_3_)_3_·9H_2_O (0.0525 M) was titrated with 1.2 M NaOH solution until pH 9.5 at N_2_ atmosphere. The obtained slurry was located in a Teflon-lined stainless steel bomb and then reacted at 150 °C in an oven for 24 h. After centrifugation, washing, and lyophilization, pristine LDH (Mg_3_Al-NO_3_-LDH) powder was acquired and it was directly utilized for ion exchange reaction. For reconstruction, the pristine LDH was calcined to layered double oxide (LDO). The calcination process was carried out at 400 °C for 9 h by using an electric muffle furnace [28].

For the preparation of GM-LDH hybrid by ion-exchange route (GML-I), 2.22 g of Mg_3_Al-NO_3_-LDH powder was dispersed in 91.2 mL of decarbonated water and 9.8 mL of GM/DMSO solution (43 mg solid content) was added. In order to proceed the hybridization through reconstruction (GML-R), the same amount of GM/DMSO solution was mixed with 91.2 mL of decarbonated water and then 1.00 g of LDO was added. Both suspension mixtures were stirred for 24 h at room temperature in the dark. After 24 h, the powder was acquired using centrifugation, washing, and lyophilization. 

### 2.4. Characterization

In order to identify structure of pristine Mg_3_Al-NO_3_-LDH, LDO and both hybrids, we utilized an X-ray diffractometer (PXRD, SmartLab, Rigaku, Akishima-shi, Tokyo, Japan) with Cu K radiation (λ = 1.5406 Å). The powder XRD patterns were measured in the 2θ range 5°–80° with scanning rate 0.02°. The chemical formula of GML-I and GML-R were calculated by CHNS elemental analysis (EA, EA3000, Eurovector, Pavia, Italy) and thermogravimetric analysis (TGA, SDT Q600, TA Instruments, New Castle, DE, USA). The DZ content in GML-I and GML-R was quantified using HPLC with same condition as described in experimental Section 2.2. The particle size and surface morphology of pristine LDH, LDO, and both hybrids were observed using field emission-scanning electron microscope (FE-SEM, JSM-7100F, ZEOL, Akishima-shi, Tokyo, Japan). The powder sample was directly attached on carbon tape and then images were obtained with 15 kV acceleration voltage after Pt/Pd sputtering for 60 s. Zeta potential values of samples were examined by ELSZ-1000 (Otsuka, Kyoto, Japan). For measuring zeta-potential, 10 mg of each sample was dispersed in 10 mL of deionized water and then pH of the sample suspension was controlled to ~7.

### 2.5. Release Test

In order to obtain time-dependent release of DZ from both hybrids, 50 mg of each hybrid was dispersed in 100 mL media such as pH 7.4 PBS and pH 4.5 HBSS. Then, an aliquot at each time point (5, 10, 15, 25, 360, 720, 1440 min) was collected by centrifugation and filtration of 1 mL of supernatant. The obtained aliquot was quantified by HPLC and the experiments were triplicated. The time-dependent release profile was fitted by three kinds of kinetic models such as the Elovich equation (1), power function (2), and parabolic diffusion (3) for further quantitative analysis and confirming location of GM moieties in hybrid [34,35,36].

The Elovich equation describes bulk surface diffusion and release equation is written as follows

Q_t_ = *a* + *b* ln t. (1)

*a*: related quantity in the initial phase, *b*: release rate.

Power function represents drug release mediated by ion-exchange reaction. This model can be written as

Q_t_ = αt^β^. (2)

α: initial desorption rate constant, β: desorption rate coefficient.

Parabolic function indicates that drug release in interlayer of carrier is controlled by intraparticle diffusion, and equation is denoted as

Q_t_ = Q_e_ + Rt^1⁄2^. (3)

R: diffusion rate constant.

For all models, Q_t_ (µg/mg) and Q_e_ (µg/mg) stand for released amount at time t min and at equilibrium, respectively.

### 2.6. Cellular Tests

#### 2.6.1. Cytotoxicity

Human osteoblast-like MG-63 cells were cultured in DMEM media containing 10% FBS and 1% penicillin-streptomycin using CO_2_ incubator with 5% CO_2_ at 37 °C. After MG-63 cells reached 80% confluency, cells were seeded at a density of 5.0 × 10^3^ cells per well on 96-well cell culture plates. After 24 h, the GM solution and hybrid suspensions were prepared in DMEM media to have DZ concentration from 0.03 to 0.0003 µg/mL. The DMSO content in GM was set to below 2% following the cellular assay protocol. After treatment of the extract itself or hybrids, cells were incubated for 72 h. Then, cells were washed with PBS and WST-1 reagent was added. The cells were incubated at 37 °C under CO_2_ condition for 4 h. The absorbance at 437 nm was then measured using microplate reader (VARIOSKAN LUX, ThermoFisher SCIENTIFIC, Waltham, MA, USA). 

#### 2.6.2. Alkaline Phosphatase Activity

For alkaline phosphatase (ALP) activity test, MG-63 cells were seeded at a density of 1.0 × 10^5^ cells per well on six-well cell culture plates. After 24 h, the GM solution and hybrids suspensions were prepared in DMEM media to have a DZ concentration from 0.03 µg/mL. The samples were treated to cells and then the cells were incubated for 72 h. The cells were washed with PBS to remove any un-reacted samples and treated with trypsin-EDTA to detach cells from the cell culture plate. Cell pellets which were obtained by centrifugation at 2000 rpm for 2 min were washed with PBS to eliminate residual trypsin-EDTA. In order to lyse cells, the pellets were mixed with 0.5% triton-X100 containing PBS and cell suspension was subjected to a freezing and thawing process. Supernatants of cell lysate were obtained using centrifugation and then 100 μL of cell supernatant was mixed with 200 μL p-NPP solution for 30 min at 37 °C using an agitator. Then, absorbance of each mixture was measured using a microplate reader with a wavelength of 405 nm. For normalization of ALP activity results on the basis of protein concentration, we conducted the Bradford assay. Supernatants of the cell suspension were mixed with protein assay dye reagent. After shaking for 5 min by orbital shaker (SH30, FINEPCR, Gunpo-si, Gyeonggi-do, South Korea), protein concentration was quantified by measuring absorbance of the mixture using a microplate reader with a wavelength of 595 nm. 

#### 2.6.3. SEM Images of Collagen Fiber Exposed from MG-63 Cells

MG-63 cells were cultured on slide glass and then samples were treated with the same procedure of ALP activity assay. After 72 h, cells were immobilized and dehydrated using 4-paraformaldehyde and a concentration-gradient of ethanol (30%, 50%, 70%, 80%, 90%, and 100%), respectively, and then dried after treatment with hexamethyldisilazane. The SEM image of collagen fiber around MG-63 cells was acquired by FE-SEM after Pt/Pd sputtering for 60 s.

## 3. Results and Discussion

**Figure 1:**
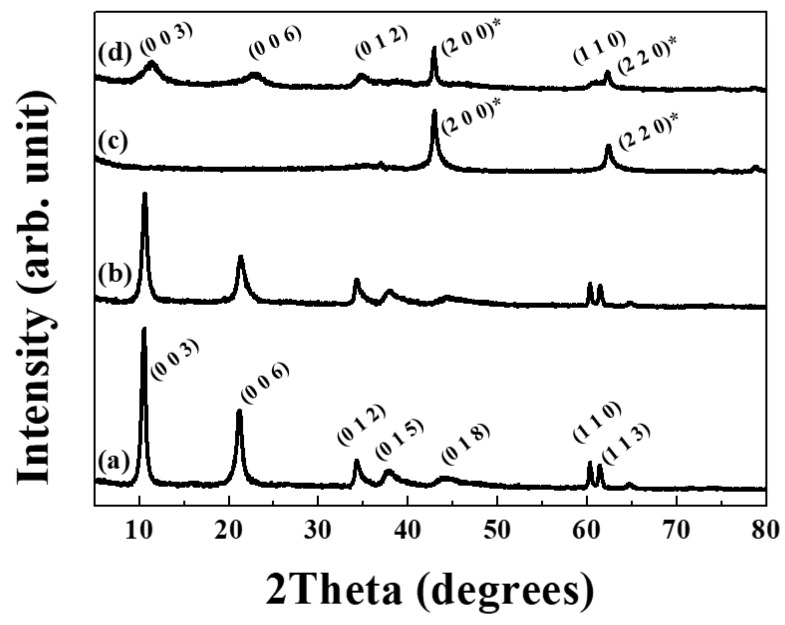
Powder XRD patterns of (**a**) pristine layered double hydroxide (LDH), (**b**) *Glycine max Merrill* Extract (GM)-LDH hybrid prepared by ion-exchange (GML-I), (**c**) layered double oxide (LDO), and (**d**) GM-LDH hybrid by reconstruction (GML-R). Asterisks indicate the peak of periclase (MgO) phase.

The crystal structures of the pristine Mg_3_Al-NO_3_-LDH, LDO, GML-I, and GML-R were investigated by XRD patterns. The pristine LDH showed characteristic peaks of hydrotalcite-like (Joint Committee on Powder Diffraction Standards (JCPDS) No. 14-0191) at 10.56°, 21.18°, 34.25°, 37.71°, 43.81°, 60.36°, and 61.39° for (003), (006), (012), (015), (018), (110), and (113) peaks, respectively (Figure 1a) [37]. The d-spacing of pristine LDH calculated from the (003) peak was 8.37 Å, which well matched with that of previously reported NO_3_^−^ intercalated LDH [37]. After GM incorporation into LDH by ion-exchange reaction, a similar XRD pattern with peaks at 10.60°, 21.29°, 34.25°, 37.83°, 44.43°, 60.32°, and 61.45° for (003), (006), (012), (015), (018), (110), and (113) reflection, respectively, was found (Figure 1b). The d-spacing of GML-I, 8.34 Å, was similar with that of pristine LDH, indicating that intercalation of GM moieties into the interlayer space of LDH did not occur. Although there was no significant difference in crystalline phase, we could observe change in crystallinity before and after GM adsorption by ion exchange. The crystallinity along the c-axis and ab-plane directions were calculated by Scherrer’s equation (t = 0.9λ/Bcosθ, t: crystallite size (Å), λ: X-ray wavelength, B: full-width at half-maximum of peak, θ: Bragg angle). The crystallite size along (003) reduced from 15.8 to 13.4 nm after ion exchange; that along (110) was fairly similar before and after the reaction showing 29.6 and 28.8 nm, respectively. Similar results of crystallinity reduction along the c-axis were reported previously when berberine chloride, lactate dehydrogenase, and alkaline phosphatase were adsorbed just on the surface of LDH [19,38,39].

During calcination, LDH lost interlayer water and anions and hydroxides and then transformed to layered double oxide containing MgO, periclase (JCPDS No. 87-0653), and amorphous Al_2_O_3_, which showed a characteristic (200) and (220) peak at 62.36° and 42.95° (Figure 1c) [40]. When the reconstruction reaction was over, pristine LDH phase was recovered by exhibiting characteristic (003), (006), (012), and (110) peaks at two theta values 11.38°, 22.53°, 34.74°, and 60.96°, respectively (Figure 1d). There was periclase phase still observed with (200) and (220) peaks at 42.93° and 62.29°, respectively; however, the existence of periclase would not have a negative effect on the current GML-R hybrid in terms of encapsulation, release control, and biological activity of GM as the periclase is bio-compatible and bio-inert [41,42]. Upon the reconstruction reaction, crystallite size of LDH significantly decreased from 15.8 and 29.6 nm to 3.69 and 8.72 nm corresponding to (003) and (110) peaks, respectively. The severe crystallinity change is thought to be due to large molecular dimension of GM moiety. In our previous report, incorporation of *Angelica gigas* Nakai natural extract into LDH through reconstruction significantly reduced crystallinity of LDH due to perturbed stacking of LDH layers by the steric hindrance of large organic species in natural extract [28].

**Figure 2:**
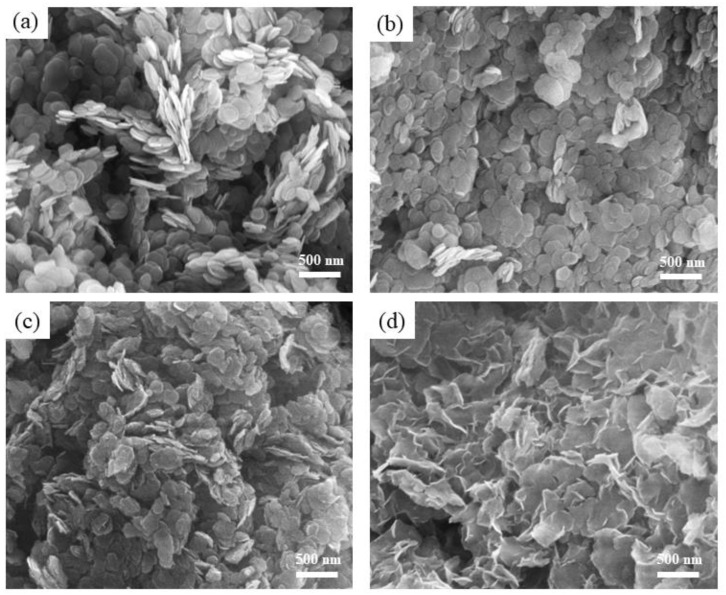
SEM image of (**a**) pristine LDH, (**b**) GML-I, (**c**) LDO, and (**d**) GML-R.

In order to compare particle morphology and size of LDH depending on incorporation method, we investigated SEM images of pristine LDH, LDO, GML-I, and GML-R hybrids (Figure 2). The pristine LDH particles showed a coin-like shape with smooth surface and the particle size was 154 ± 33.6 nm (Figure 2a). After ion-exchange reaction, there was no significant change in terms of particle size and morphology: coin-like structure with smooth surface and lateral size of ~159 ± 26.3 nm (Figure 2b). Through XRD patterns and SEM images, we suggested that GM moieties would attach on surface of GML-I rather than intercalate in the interlayer space of LDH. In the reconstruction reaction, the calcined LDH, i.e., LDO showed similar particle size (159 ± 27.9 nm) with pristine LDH (Figure 2c). It was worthy to note that the GML-R hybrid, completely different from GML-I, showed house-of-cards like structure (Figure 2d), suggesting that particle thickness of LDH becomes thinner and that the layers were randomly stacked with GM moiety. This morphology tends to have inter-particle space, which might encapsulate large biomolecules in natural extract as reported previously [28]. We could observe a few LDO-like particles in the SEM image of GML-R which corresponded to the XRD result. Nonetheless, the majority house-of-cards morphology is expected to provide GM moieties with inter-particle space as reservoir. As the XRD and SEM showed different crystallographic and morphological features for GML-I and GML-R, we expected that the different incorporation method influenced status of the extract in terms of encapsulation and release.

The existence of GM moieties including bioactive component daidzein (DZ) was confirmed with HPLC measurement of both GML-I and GML-R dissolved solution (Figure S2). The amount of GM loading was determined with EA and TGA results (Figure S1). The content of water and remaining metal oxide were also obtained from the TGA curve (Figure S1). Based on the stoichiometry, GM loading amount, water, and remaining metal oxide contents, chemical formulae were determined: [Mg_3_Al(OH)_8_(NO_3_^−^)](GM)·1.9H_2_O and [Mg_3_Al(OH)_8_(CO_3_^2−^)_0.5_(GM)·4.5H_2_O][Mg_3_AlO_4.5_]_0.82_ for GML-I and GML-R, respectively. The loading capacity of GM for GML-I and GML-R calculated from chemical formula was 15.6% and 18.6%, respectively. Unexpectedly the loading capacity of GM was not significantly affected by the incorporation method. Furthermore, the encapsulation efficiency of GM was not different between GML-I and GML-R (Table 1). These results implied that the natural extract loading by LDH had a certain limitation. In fact, our previous research on *Angelica gigas* Nakai extract incorporation into LDH showed similar loading capacity of 18.4% [28]. 

Although the loading capacity was similar regardless of incorporation method, there clearly was observed incorporation selectivity on certain molecules. The major bioactive substances in GM are isoflavonoid derivatives like DZ which is a representative species affecting bone regeneration [43]. The encapsulation efficiency and loading capacity of DZ were quantitatively investigated by HPLC. As shown in Table 1, the encapsulation efficiency for DZ was fairly different from that of GM itself. The ion exchange sample showed lower encapsulation efficiency of DZ (53.7%) than GM (67.2%); on the other hand, encapsulation of DZ (98.1%) was much more efficient in reconstruction than GM (65.1%). Consequently, the loading capacity of DZ showed difference according to the incorporation methods, 0.0108% and 0.0286% for GML-I and GML-R, respectively. The results strongly suggested that the reconstruction method selectively encapsulate the DZ moiety by LDHs. A similar result was observed in our previous report on *Angelica gigas* Nakai extract encapsulation. Biologically active species, decursin in the total extract was enriched after hybridization [28]. In order to quantitatively analyze the selectivity, we calculated DZ content in total extract as shown in Table 1. The GM itself contained 0.095% of DZ in it. When GM was incorporated into LDH by ion exchange, there existed 0.076% of DZ in the GM portion; however, reconstruction concentrated it up to 0.143%. It is not clear at this stage why reconstruction had selectivity on DZ encapsulation compared with other species in the GM. Nevertheless, this result suggested that DZ-LDH attraction possibly through dipole-charge interaction or hydrogen bonding became more facilitated upon reconstruction reaction.

**Figure 3:**
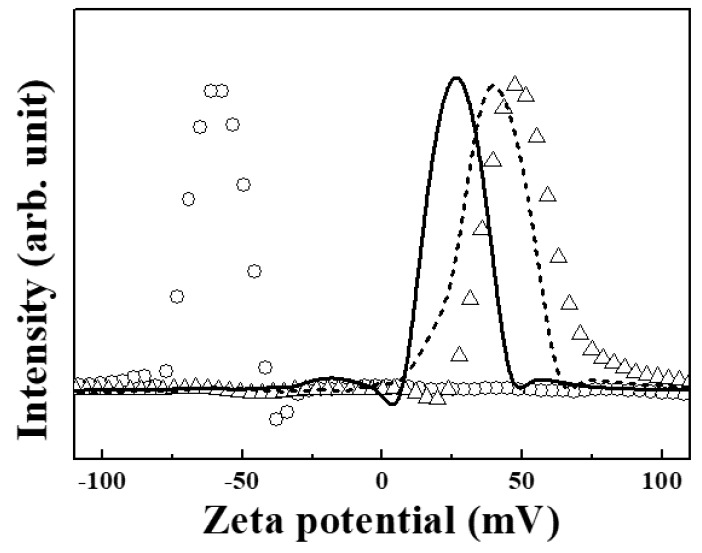
Zeta potential of GM itself (open circle), pristine LDH (open triangle), GML-I (solid line), and GML-R (dashed line).

**Scheme 1:**
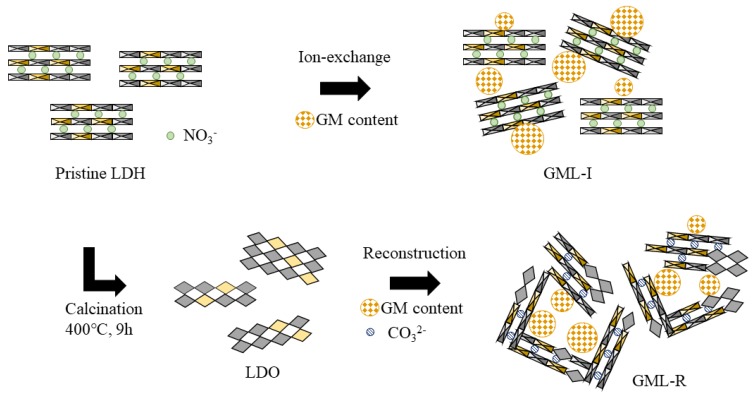
Schematic diagram of incorporation reaction between GM and LDH by ion-exchange and reconstruction process.

In order to investigate the location of GM in both GML hybrids, we measured zeta potential of GM, pristine LDH and both hybrids at pH 7 aqueous media. Figure 3 indicates that pristine LDH and GM showed clear positive and negative potential 46.9 ± 1.57 mV and −56.6 ± 1.81 mV, respectively (Figure 3 open triangle and circle). The zeta potential of GML-I and GML-R were between that of GM and LDH, but more close to that of LDH exhibiting 26.1 ± 2.01 mV and 42.4 ± 2.01 mV, respectively (Figure 3 solid line and dashed line). This result suggested that the outer-sphere of hybrid had more LDH character than GM; i.e., the inorganic part (LDH) effectively encapsulated the organic part (GM). Between GML-I and GML-R, the latter showed more positive zeta potential than the former. Thus, we could expect that GML-R was better than GML-I in terms of safe preservation of extract moiety. From XRD patterns, SEM images, zeta-potential values, the incorporation process could be described as Scheme 1. In other words, GM moieties adsorbed on LDH surface by dipole-charge interaction or hydrogen bonding maintaining their interlayer anion through ion-exchange process. In contrast, during reconstruction reaction, calcined LDH recovered their layered structure with carbonate as interlayer anion. At the same time, GM moieties incorporated in inter-particle space formed by recovered LDH and remaining LDO.

**Figure 4:**
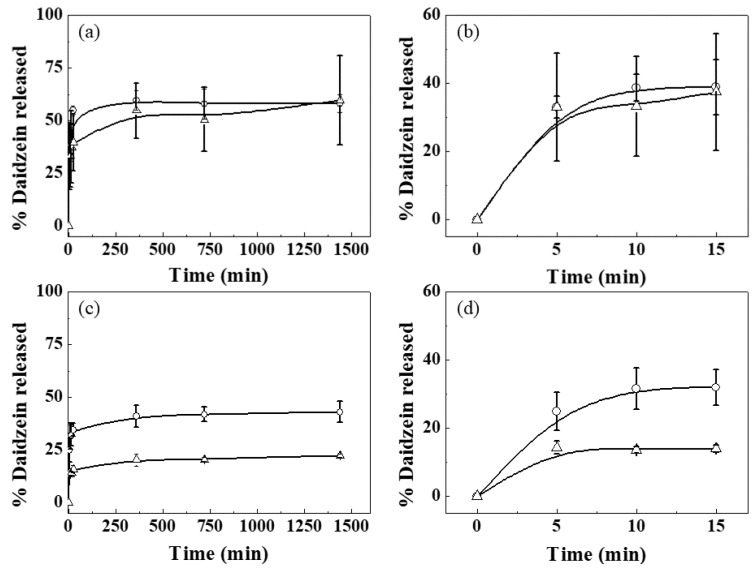
Time-dependent release patterns of DZ from GML-I for (**a**) overall time, (**b**) initial time and from GML-R for (**c**) overall time and (**d**) initial time in pH 4.5 Hank’s balanced salt solution (HBSS) condition (open circle) and pH 7.4 phosphate buffered saline (PBS) condition (open triangle).

As we confirmed that the state of encapsulated GM was different depending on synthetic route, we further investigated time-dependent DZ release from hybrids. Figure 4 shows release profiles of DZ from both hybrids in neutral physiological condition (pH 7 PBS) and simulated lysosomal condition (pH 4.5 HBSS). In early stage, DZ was released fast from GML-I showing cumulative release of 38.8% in pH 7.4 and 37.5% in pH 4.5 within 15 min. The DZ release from GML-I reached equilibrium during 24 h with 58.1% and 59.8% of cumulative release in pH 7.4 and pH 4.5, respectively. In GML-I, there was no significant difference in DZ release with respect to release media, because surface adsorbed DZ could be easily dissolved out in aqueous media regardless of pH condition. On the other hand, DZ release from GML-R was fairly different depending on media: 14.0% in pH 7.4 and 31.9% in pH 4.5 within 15 min. Release of DZ within 15 min was suppressed 2.5 times in pH 7.4 compared with in pH 4.5 condition. The total release amount of DZ from GML-R was also different, 22.3% in pH 7.4 and 43.0% in pH 4.5 for 24 h. In the case of GML-R, the walls of house-of-cards structure, i.e., LDH layers, could be dissolved in acidic pH condition; hence DZ localized in the inter-particle pore would be easily released in pH 4.5. The pH-dependent release property of GML-R is advantageous to deliver DZ into cells and to trigger its action. In neutral physiological pH such as in culture media or plasma, the encapsulated DZ was well preserved. Once the hybrids enter the cell via endocytosis, they are transferred to the endosome and lysosome where the pH is around 4–5. The DZ moiety in GML-R would then be released to act as bone-regenerator; however, GML-I lost most of DZ even in neutral pH and thus the delivery ability of LDH cannot work effectively. 

For quantitative analysis, time-dependent release profiles were fitted to kinetic models, and the result showed that DZ release was the best matching with the Elovich equation (Table 2) in all tested conditions. The Elovich model hypothesizes that the release of encapsulated moiety is mediated by bulk surface diffusion. Therefore, DZ release was influenced by the interaction between DZ molecules and bulk surface of LDH particles in GML-I or GML-R. The *a* and *b* parameters of the Elovich equation were calculated from the linear regression result of release patterns (Table 3). The Elovich parameter *a* is associated with the released amount in initial time; the higher *a* value, the more amount is released at the initial stage [44]. The parameter *b* is related to the release rate of a loaded content [35]. GML-I indicated similar *a* and *b* values, regardless of pH: *a* values 18.40 and 14.77 and *b* values 2.09 and 2.14 in pH 7.4 and pH 4.5, respectively. The result quantitatively confirmed that GML-I released DZ in a non-pH-selective manner. On the other hand, GML-R showed clear pH dependence; both *a* and *b* values in pH 4.5 were bigger than those in pH 7.4 condition. According to different *a* and *b* values, we could expect that DZ release was fairly suppressed in the neutral condition but facilitated in lysosomal pH. From the release profile, we suggested that the reconstruction method was more effective than ion-exchange reaction for natural extract moiety delivery.

As we mentioned in the introduction, GM has biological activity in bone-regeneration. Especially, DZ is well known to promote osteoblast differentiation in cells like MG-63 by increasing ALP activity [45,46]. The enzyme, ALP, helps mineralization of calcium phosphate on collagen fiber and thus increasing ALP activity leads to effective osteoblast differentiation [43]. In this sense, we investigated ALP activity against human osteoblast-like MG-63 cells utilizing GM and hybrids under the same DZ concentration.

**Figure 5:**
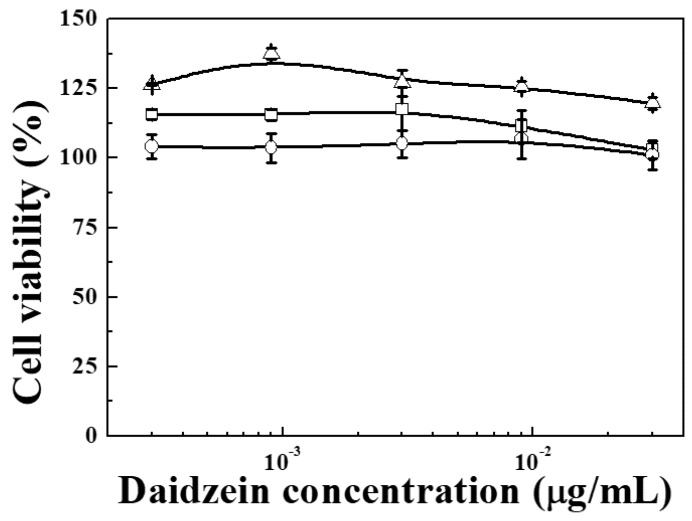
Cytotoxicity of GM (open square), GML-I (open circle), and GML-R (open triangle) against the MG-63 cell line.

Before ALP activity assay, we first checked cytotoxicity of GM, GML-I, and GML-R hybrids against human MG-63 cells. As shown in Figure 5, GM, GML-I, and GML-R hybrids showed more than 100% cell viability in the DZ concentration range from 0.0003 to 0.03 μg/mL. This result indicated that all the samples were biocompatible at DZ concentrations up to 0.03 μg/mL which was selected as the administration concentration for ALP activity test. 

**Figure 6:**
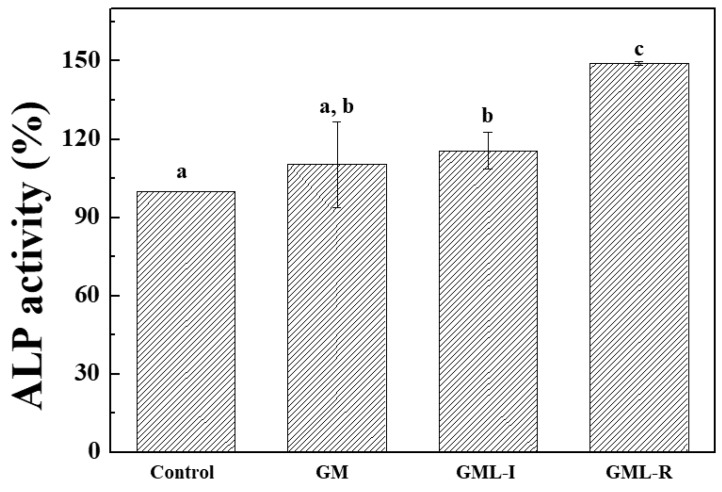
Alkaline phosphatase (ALP) activity of control (untreated), GM, GML-I, and GML-R treated MG-63 cells. The alphabet notation stands for the statistical equivalence and difference with confidence intervals of 98% calculated by Student’s *t*-test. The same alphabet indicates same APL activity between two groups.

**Figure 7:**
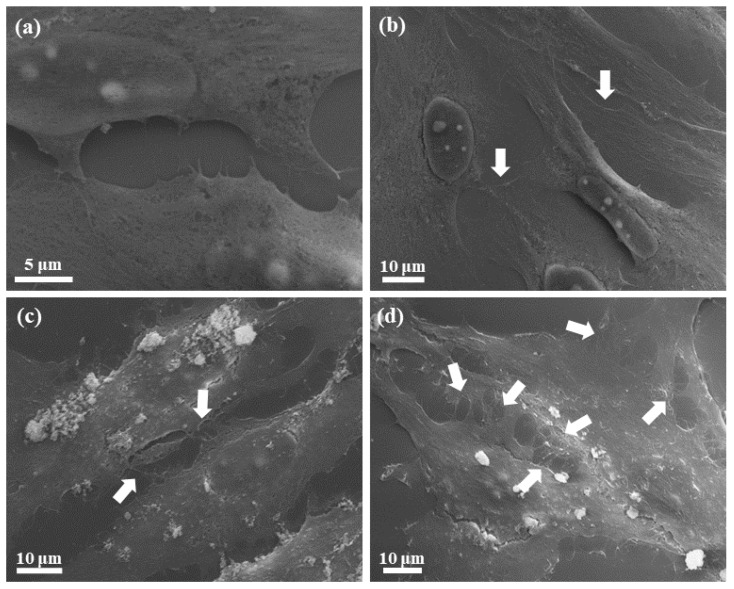
SEM image of (**a**) control (untreated), (**b**) GM, (**c**) GML-I, and (**d**) GML-R treated MG-63 cells for 72 h. Arrows stand for the formation of collagen.

For comparison, non-treated MG-63 cells were used as control (100%) and the ALP activity effect of GM, GML-I, and GML-R were compared. As displayed in Figure 6, ALP activity of GM itself was 110.3% ± 16.4%. Although GML-I had 115.5% ± 7.11% of ALP activity, the values were not statistically different from that of GM itself. It was noteworthy that GML-R showed significantly enhanced ALP activity of 149.0% ± 0.61%. Those results suggested that not only the cellular uptake property of LDH but also pH dependent DZ release of GML-R promoted ALP activity effectively. In terms of bone regeneration, isoflavone increases collagen fiber formation as well as ALP activity; collagen fibers are formed and then mineralization of calcium phosphate occurs on the fiber upon the action of ALP [39]. The collagen fiber formation by GM and both GML hybrids was investigated by SEM. Figure 7 showed MG-63 cells with or without treatment of GM, GML-I, and GML-R. After 72 h, untreated MG-63 cells showed relatively clean features between cells (Figure 7a). A few collagen fibers, which connected cells, were observed in the SEM image of GM or GML-I treated MG-63 cells (Figure 7b,c). On the other hand, much more collagen fibers were found in GML-R treated MG-63 cells (Figure 7d) than the others. This results also corresponded with ALP activity of GM and both hybrids, which suggested that the reconstruction method was the more effective way to encapsulate natural extract containing bioactive substances in LDH as drug delivery carriers than ion-exchange reaction.

## 4. Conclusions

We prepared a hybrid of soybean extract (GM) and LDH by two different methods, ion-exchange and reconstruction. According to XRD patterns and SEM images, we confirmed that the GML-I hybrid contained GM moiety on the surface of LDH particles and that GML-R made an inter-particle cavity to accommodate extract components. Zeta-potential values showed more positive charge of GML-R than GML-I which indicated more LDH character of the surface of the GML-R hybrid having the advantage of preservation of extract moiety. Quantitative analyses on GM and DZ incorporation showed that the reconstruction method selectively encapsulated DZ into LDH compared with the ion-exchange method, while the total GM loading amount was not affected by the incorporation method. Time-dependent DZ release profile well matched with the Elovich equation indicating bulk surface diffusion governed DZ release from hybrids. The result revealed that GML-I showed a similar release pattern regardless of pH condition; however, GML-R exhibited pH dependent release of DZ due to difference in GM encapsulation status. For investigating the influence of preparation methods on biological activity of hybrids, we evaluated ALP activity on MG-63 cells at non-cytotoxic concentration. The GML-R indicated 1.5 times enhanced ALP activity compared with the others, due to the pH dependent DZ release property and cellular uptake property of LDH. The formation of collagen fiber, which was increased by DZ and affected mineralization, also increased on GML-R treated MG-63 cells. From these results, we demonstrated that the reconstruction method was effective for preparing extract-LDH as a drug delivery system.

## Figures and Tables

**Table 1:** Loading capacity and encapsulation efficiency of GM and daidzein (DZ) in hybrids, and DZ content in total extract moiety in GM, GML-I, and GML-R.

	GM Encapsulation Efficiency (%)	DZ Encapsulation Efficiency (%)	GM Loading Capacity (GM/Hybrid (wt/wt%))	DZ Loading Capacity (DZ/Hybrid (wt/wt%))	DZ Content in Total Extract ^a^ (DZ/Extract (wt/wt%))
GM	-	-	-	-	0.095
GML-I	67.2	53.7	15.6	0.0108	0.076
GML-R	65.1	98.1	18.6	0.0286	0.143

^a^ (weight of DZ/weight of GM) × 100%.

**Table 2:** Kinetic model fitting results of time-dependent DZ release from GML-I and GML-R in pH 7.4 PBS and pH 4.5 HBSS.

		Kinetic Model	Regression Equation	R^2^
In pH 4.5 HBSS	GML-I	Elovich equation	Q_t_ = 2.1388lnt + 14.768	0.7795
		Power function	lnQ_t_ = 0.0943lnt + 5.7638	0.7632
		Parabolic diffusion	Q_t_ = 0.0049t^1/2^ + 0.4323	0.4217
	GML-R	Elovich equation	Q_t_ = 4.0052lnt + 33.72	0.9297
		Power function	lnQ_t_ = 0.081lnt +5.5141	0.8820
		Parabolic diffusion	Q_t_ = 0.0032t^1/2^ + 0.3216	0.4955
In pH 7 PBS	GML-I	Elovich equation	Q_t_ = 2.088lnt + 18.402	0.9550
		Power function	lnQ_t_ = 0.1037lnt + 5.6246	0.9546
		Parabolic diffusion	Q_t_ = 0.0059t^1/2^ + 0.3750	0.6509
	GML-R	Elovich equation	Q_t_ = 2.2391lnt + 15.139	0.9621
		Power function	lnQ_t_ = 0.0897lnt + 4.7521	0.9563
		Parabolic diffusion	Q_t_ = 0.0021t^1/2^ + 0.147	0.7426

**Table 3:** *a* and *b* parameters of the Elovich equation for fitting result of GML-I and GML-R in pH 4.5 HBSS and in pH 7.4 PBS condition.

		*a*	*b*
GML-I	pH 7.4 PBS	18.40	2.09
	pH 4.5 HBSS	14.77	2.14
GML-R	pH 7.4 PBS	15.14	2.24
	pH 4.5 HBSS	33.72	4.01

## Supplementary Materials

The Supplementary Materials are available online at https://www.mdpi.com/2079-4991/9/9/1262/s1.
