# Supplementary material for: Incorporation of Glycine max Merrill Extract into Layered Double Hydroxide through Ion-Exchange and Reconstruction
Source: Nanomaterials (Basel). 2019 Sep 5;9(9):1262. doi: 10.3390/nano9091262 (PMC6781017; doi:10.3390/nano9091262)
Supplement: Supplementary file 1 [file nanomaterials-09-01262-s001.pdf]

Supporting Information

Incorporation of *Glycine max Merrill* Extract into Layered Double Hydroxide through Ion-Exchange and Reconstruction

Do-Gak Jeung, Hyoung-Jun Kim* and Jae-Min Oh*

Department of Energy and Materials Engineering, Dongguk University-Seoul, 04620 Seoul, South Korea

* Corresponding author, email: hjun.kim@dongguk.edu (H.-J.K); jaemin.oh@dongguk.edu (J.-M.O)

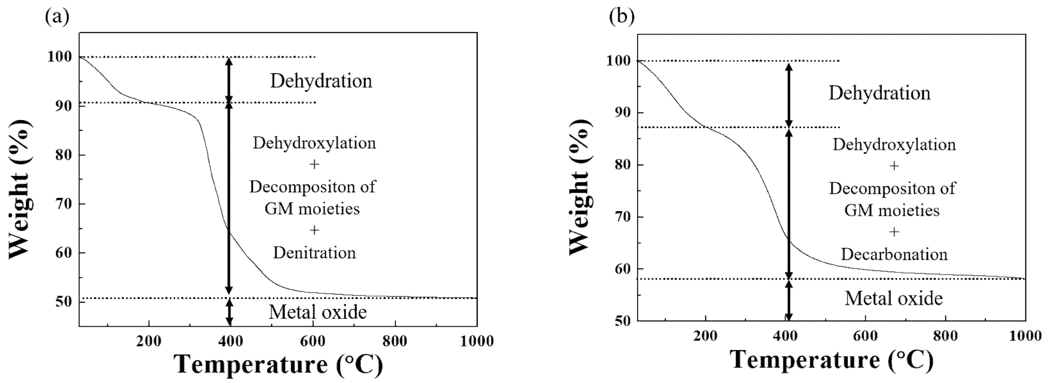

Figure S1. Thermogravimetric analysis of (a) GML-I and (b) GML-R hybrid.

Supporting Information

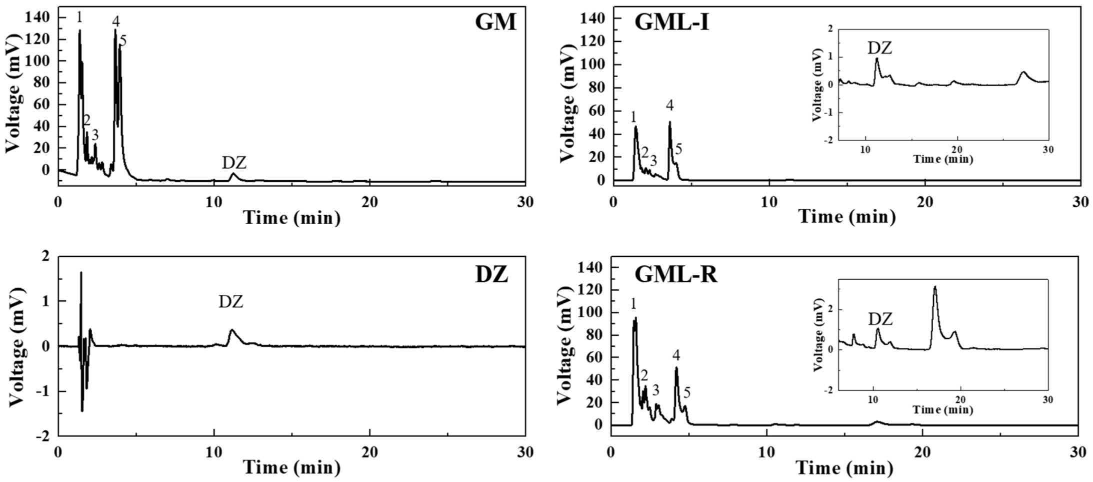

Figure S2. HPLC chromatogram of GM, DZ, GML-I and GML-R hybrid.
